# The prognostic role of food addiction for weight loss treatment outcomes in individuals with overweight and obesity: A systematic review and meta‐analysis

**DOI:** 10.1111/obr.13851

**Published:** 2024-10-16

**Authors:** Georg Halbeisen, Marie Pahlenkemper, Luisa Sabel, Candice Richardson, Zaida Agüera, Fernando Fernandez‐Aranda, Georgios Paslakis

**Affiliations:** ^1^ University Clinic for Psychosomatic Medicine and Psychotherapy, Medical Faculty, Campus East‐Westphalia, Ruhr‐University Bochum Luebbecke Germany; ^2^ Temerty Faculty of Medicine University of Toronto Toronto Canada; ^3^ Departament d'Infermeria de Salut Pública, Salut Mental i Materno‐Infantil, Escola d'Infermeria, Facultat de Medicina i Ciències de la Salut Universitat de Barcelona Barcelona Spain; ^4^ Research Group in Mental health, Psychosocial and Complex Nursing Care (NURSEARCH), Facultat de Medicina i Ciències de la Salut Universitat de Barcelona Barcelona Spain; ^5^ CIBER Fisiopatología Obesidad y Nutrición (CIBERobn) Instituto de Salud Carlos III Madrid Spain; ^6^ Psychoneurobiology of Eating and Addictive Behaviors Group, Neurosciences Programme Bellvitge Biomedical Research Institute (IDIBELL), L'Hospitalet de Llobregat Barcelona Spain; ^7^ Eating Disorders Unit, Clinical Psychology Unit University Hospital of Bellvitge L'Hospitalet de Llobregat Spain; ^8^ Department of Clinical Sciences, School of Medicine and Health Sciences University of Barcelona Barcelona Spain

**Keywords:** bariatric surgery, diverse populations, food addiction, gender differences, obesity, psychotherapy, weight loss

## Abstract

Food addiction (FA) could be a potential prognostic factor of weight loss intervention outcomes. This systematic review with meta‐analysis aimed to (1) estimate this prognostic effect of FA diagnosis and symptom count in individuals with overweight or obesity and (2) explore potential sources of heterogeneity based on properties of the weight loss intervention, study, and sample (e.g., age, gender, ethnicity). We searched PubMed, PsycINFO, and Web of Science for studies reporting on associations between pre‐intervention FA (assessed with the Yale Food Addiction Scale) and weight outcomes after weight loss intervention in individuals with overweight or obesity without a medically diagnosed eating disorder. Twenty‐five studies met inclusion criteria, including 4904 individuals (71% women, *M*_age_ = 41 years, BMI = 40.82 kg/m^2^), *k* = 18 correlations of weight loss with FA symptom count, and *k* = 21 mean differences between FA diagnosis groups. Pooled estimates of random‐effects meta‐analyses found limited support for a detrimental effect of FA symptom count and diagnosis on weight loss intervention outcomes. Negative associations with FA increased for behavioral weight loss interventions and among more ethnically diverse samples. More research on the interaction of FA with pre‐existing mental health problems and environmental factors is needed.

## 1 BACKGROUND

Phenotypical and neuroendocrinological similarities between addiction and obesity (OB) led to the proposal of “food addiction” (FA),^1^, ^2^ addictive‐like eating that is characterized by a loss of control over food intake, overconsumption of hypercaloric foods to cope with problems, and an intense and persistent craving for highly palatable foods.^3^ Despite increasing interest in the idea that FA may contribute to the development of OB, the concept remains controversial.^4^ Open questions include whether FA should be considered a substance‐based or behavioral addiction,^5^ its nosological status when compared to eating disorders,^6^ and its clinical implications.^7^ To further the discussion, the present systematic review with meta‐analysis assessed the prognostic role of FA for weight loss treatment outcomes in individuals with OB.

FA, though not currently recognized as a formal disorder, is typically assessed with (variants of) the Yale Food Addiction Scale (YFAS),^3^ which adapted the diagnostic criteria for substance dependence to consuming high‐calorie foods. Specifically, the YFAS includes 25 items that capture the seven substance dependence criteria specified in the Diagnostic and Statistical Manual of Mental Disorders, Fourth Edition, Text Revision (DSM‐IV‐TR),^8^ including loss of control over consumption, unsuccessful attempts of quitting, and withdrawal symptoms. The YFAS offers two scoring options: a symptom count (0 to 7) and a dichotomous diagnosis (“positive,” if three or more criteria are met in addition to significant clinical impairment vs. “negative”). Variants include adaptations for children (YFAS‐C),^9^ a modified nine‐item version (mYFAS),^10^ and the updated 35‐item YFAS 2.0,^11^ to reflect the merging of seven substance dependence criteria with four substance abuse symptoms in the DSM‐5.^12^ The YFAS 2.0 symptom counts thus range from 0 to 11, and the positive diagnoses (starting at two instead of three symptoms) are further stratified into mild, moderate, and severe FA. Both the original and 2.0 variants of the scale show positive associations with body weight and disordered eating^7^, ^13^ and are available in multiple languages.^14^

Prevalence rates for FA based on YFAS among individuals with OB range from 7.7 to 47%,^15^ with a recent systematic review and meta‐analysis pointing to a 30% weighted average.^16^ Pronounced FA rates are found in individuals with eating disorders (55% to 84%)^16^ and among women in some reviews^17^, ^18^, ^19^; potential effects of other sociodemographic characteristics, including age, require further exploration.^16^ The presence of FA in individuals with OB has been associated with greater psychopathology in terms of depressive and anxiety symptoms^20^ and poorer life quality.^21^ FA has also been associated with weight gain and weight‐control failure in individuals with OB,^22^, ^23^ raising speculations about FA as a potential prognostic factor and intervention target of OB treatment outcomes.^24^

Indeed, systematic reviews identified substantial variation in weight loss after OB treatment,^25^, ^26^, ^27^ with some estimates suggesting that only 20% to 30% of initial weight loss can be maintained.^28^ Effective treatment options for OB include behavioral weight loss strategies (e.g., dieting, exercise), weight loss (i.e., bariatric) surgery, pharmacotherapy, and psychotherapy such as cognitive‐behavioral approaches.^29^, ^30^ Few studies have investigated the prognostic role of FA for OB treatment outcomes among adults and adolescents, but recent narrative reviews suggest the available evidence is mixed.^31^ Among individuals with OB without co‐morbid eating disorders (e.g., binge‐eating disorder), pre‐intervention FA diagnosis and increased FA symptom count predicted lower weight loss after bariatric surgery and behavioral weight loss interventions in some studies.^32^, ^33^, ^34^, ^35^ Other studies observed no associations between pre‐intervention FA and weight loss.^36^, ^37^, ^38^ These heterogeneous findings stem from studies that vary widely in sample size, the type of intervention, and the post‐intervention follow‐up; thus, systematic investigations of the relation between pre‐intervention FA and weight loss outcomes are needed.

So far, only one systematic review addressed the relation between pre‐intervention FA in adults with OB and post‐surgical weight loss.^39^ Based on only three available studies, the review concluded that pre‐surgery FA does not predict post‐operative weight regain or weight outcomes at up to 12 months of follow‐up. Since its publication in 2017, however, several additional studies have examined the predictive role of FA for bariatric surgery and other weight loss intervention outcomes that may qualify that conclusion.^35^, ^40^, ^41^ In addition, we are unaware of any attempt to statistically synthesize observed associations between pre‐intervention FA and post‐intervention weight loss (i.e., by meta‐analysis), which could pool association estimates adjusted for sampling variability and explore potential sources of heterogeneity using meta‐regression. Therefore, the present systematic review with meta‐analysis aimed to (1) estimate the prognostic effect of FA diagnosis and symptom count, as measured by YFAS, in individuals with overweight or OB on weight outcomes following weight loss treatment and (2) explore potential sources of heterogeneity of the association based on properties of the intervention, the study, and the sample characteristics (e.g., age, gender, ethnicity).

## 2 METHODS

This systematic review and meta‐analysis protocol was prospectively registered with the PROSPERO database of systematic reviews (CRD42022384703) and follows the preferred reporting for systematic reviews and meta‐analyses (PRISMA) guidelines.^42^

### 2.1 Eligibility criteria

We included any peer‐reviewed study published in English, German, Greek, or Spanish that (1) reported on repeatedly measured weight outcomes of (2) a weight loss intervention in (3) individuals with overweight or obesity (BMI ≥ 25 kg/m^2^) without a medically diagnosed, co‐morbid eating disorder. Each study (4) must have had assessed food addiction using any variant of the YFAS before the intervention and (5) correlated pre‐intervention YFAS symptom count with weight loss outcomes (e.g., % total weight loss, % excess weight loss, delta BMI, raw weight adjusted for pre‐intervention levels) or compared weight loss outcomes between individuals with pre‐intervention FA positive and FA negative diagnoses. We excluded studies that (1) included only individuals with medically diagnosed eating disorders, (2) had an ineligible design (e.g., case reports, cross‐sectional studies, reviews, editorials), or (3) provided insufficient data to be included in the meta‐analysis.

### 2.2 Information sources

A comprehensive keyword‐based electronic database search of the peer‐reviewed literature was conducted in the online databases PubMed, PsycINFO, and Web of Science, on January 26, 2023, and updated on June 3, 2024. We searched the reference lists of included articles by hand for other relevant studies that might meet the inclusion criteria.

### 2.3 Search strategy

The keyword search included natural language terms and Medical Subject Headings (MeSH), where applicable, related to (1) food addiction (e.g., food addiction, compulsive eating, YFAS) in conjunction with (2) patient weight status (e.g., obesity, overweight) and (3) any surgical, behavioral, or pharmacological weight loss intervention (e.g., bariatric surgery, physical exercise, orlistat). The full electronic database searches and the list of keywords are provided in the Supporting Information. Duplicates were removed automatically with the Covidence systematic review software.^43^

### 2.4 Selection process

Five reviewers (L.S., G.H., M.P., C.R., Z.A.) independently screened titles and abstracts using Covidence.^43^ Three reviewers (L.S., G.H., M.P.) performed the full text screening. Two decisions were required per each title/abstract and full text. Disagreements were discussed in consensus meetings, and G.H. made the final decision for study inclusion or exclusion.

### 2.5 Data collection process and data items

Three reviewers (L.S., G.H., M.P.) independently extracted, in duplicate, the title, reference, publication year, funding sources, conflicts of interest, type of study, study design, participant information (e.g., gender, age, ethnicity), sample sizes, type of treatment, type of outcome measure, pre‐post measurement (follow‐up) intervals, and the authors´ conclusions concerning the role of FA for weight loss prediction. Correlations, means and standard deviations, or any other data available for effect size calculation (e.g., regression weights, *p*‐values) were extracted for each available timepoint and/or subgroup.

### 2.6 Study risk of bias assessment

Three reviewers (L.S., G.H., M.P.) evaluated the included studies for quality using the Cochrane risk of bias tool in non‐randomized studies and interventions (ROBINS‐I).^44^ The checklist was used to evaluate bias across seven domains (e.g., bias due to confounding, participant selection, or missing data) and to classify each study into one of five categories (low risk of bias, moderate risk of bias, serious risk of bias, critical risk of bias, no information). The assessment was completed in duplicate. For analysis, numerical values were assigned to each category, such that higher scores indicated an increased risk of bias.

### 2.7 Effect measures and synthesis methods

We used two random effects meta‐analyses with unbiased restricted maximum likelihood estimation^45^ to investigate the prognostic effect of FA on weight loss intervention outcomes in individuals with overweight and obesity. Using the available data, we extracted or computed correlations of the association between pre‐intervention YFAS symptom count and post‐intervention weight loss for the first meta‐analysis. Fisher's r‐to‐z transformation for variance stabilization was applied prior to the analysis.^46^ Correlations were coded such that negative values indicate lower weight loss with higher levels of pre‐intervention YFAS, and positive values indicate more pronounced weight loss with higher levels of pre‐intervention YFAS. The second meta‐analysis evaluated the association between pre‐intervention food addiction diagnosis (positive, negative) and post‐intervention weight loss using standardized mean differences (SMDs) between groups,^47^ which were extracted or computed using weight outcome means and standard deviations. We computed SMDs such that negative values indicate lower weight loss among individuals with FA positive compared to FA negative, whereas positive values indicate more pronounced weight loss among individuals with FA positive compared to FA negative.

In addition, for both analyses, we compared the different subgroups of weight loss interventions (e.g., bariatric surgery vs. behavioral weight loss treatment) in meta‐regression. We further explored possible effects by participant gender (% female), age (study mean), and further sociodemographic and study variables, if available (e.g., ethnicity, socio‐economic status). To account for dependent effect sizes provided by studies with multiple follow‐ups and/or multiple groups, *p*‐values and confidence intervals of model estimates were based on cluster‐robust variance estimation with studies as cluster.^48^

Statistical heterogeneity was examined using the *I* ^2^ statistic, with *I* ^2^ ≤ 40% deemed low, and *I* ^2^ ≥ 75% deemed considerable heterogeneity. For the assessment of publication biases, we inspected funnel plots with effect sizes on the horizontal axis and standard errors (SE) on the vertical axis. Egger's test was used for assessing funnel plot asymmetry.^49^ All analysis were conducted using R 4.3.3^50^ package *metafor* 4.4.0.^51^

## 3 RESULTS

### 3.1 Study selection

**FIGURE 1:**
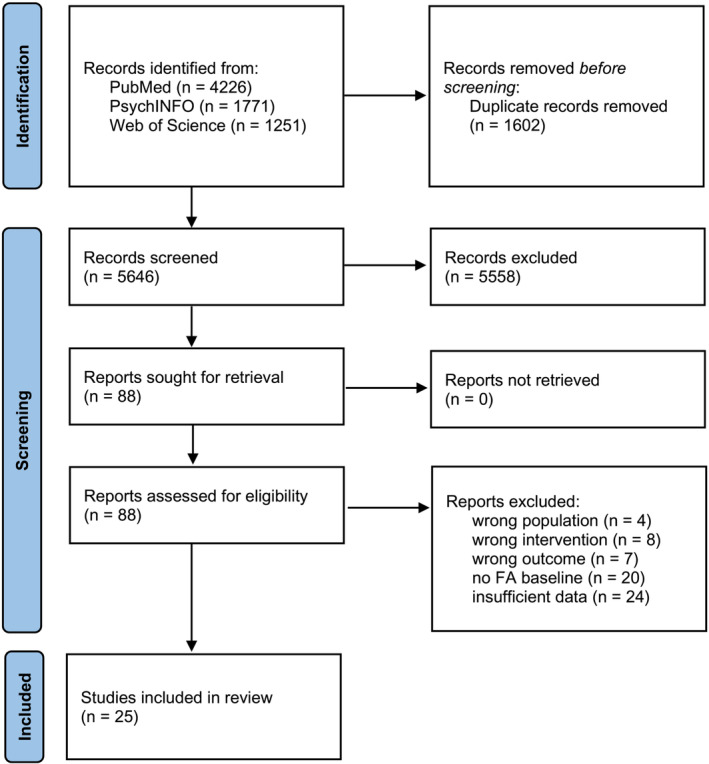
Study search and selection flow chart.

Figure 1 shows the search and selection flow chart. The database searches identified 7248 records. After removing 1602 duplicates, 5646 titles and abstracts were screened. The average rate of reviewers' agreement was 94%. After consensus discussions, 88 full texts were screened for eligibility, and 25 studies were ultimately included.

### 3.2 Study characteristics

Study details are described in Table 1. The studies were published between 2013 and 2024, with 13 conducted in the Americas,^23^, ^32^, ^34^, ^36^, ^38^, ^52^, ^53^, ^54^, ^55^, ^56^, ^57^, ^58^, ^59^ followed by eight in Europe,^22^, ^33^, ^35^, ^37^, ^41^, ^60^, ^61^, ^62^ and four in Asia.^40^, ^63^, ^64^, ^65^ Eleven studies reported findings following bariatric surgery (i.e., sleeve gastrectomy,^40^, ^41^, ^63^, ^65^ Roux‐en‐Y bypass,^56^ both,^34^, ^35^, ^38^, ^52^, ^61^ and both plus laparoscopic adjustable gastric band^57^), one following a pharmacological intervention (semaglutide^62^), and 13 following behavioral weight loss interventions (i.e., energy‐reduced diets,^23^, ^60^ physical activity promotion,^54^ a combination of both,^22^, ^33^, ^36^, ^37^, ^53^, ^55^ and interventions addressing general eating‐related attitudes and behaviors;^32^, ^58^, ^59^, ^64^ two of the latter specifically targeted addiction‐like eating behaviors^58^, ^59^). The average maximum interval between intervention and weight loss assessment was 12.53 months (SD = 7.58), and the average number of follow‐ups per study was 1.72 (SD = 0.84). The most common extracted outcome measures were percent of total weight loss (%TWL = BMI loss/[BMI‐0] × 100%, *n* = 14),^22^, ^32^, ^34^, ^35^, ^38^, ^40^, ^52^, ^53^, ^56^, ^57^, ^60^, ^61^, ^62^, ^64^ followed by raw pre‐post differences between BMI and kg (*n* = 5),^23^, ^33^, ^36^, ^37^, ^55^ and percent of excess weight loss (%EWL = BMI loss/[BMI‐25] × 100%, *n* = 3).^41^, ^63^, ^65^ Studies with children reported age‐adjusted BMI standard deviation scores (BMI‐SDS) controlled for baseline or as % change (*n* = 3).^54^, ^58^, ^59^ The YFAS versions used were YFAS^3^ (*n* = 18),^23^, ^32^, ^33^, ^34^, ^38^, ^40^, ^41^, ^52^, ^53^, ^55^, ^56^, ^57^, ^60^, ^61^, ^63^, ^64^, ^65^ YFAS 2.0^11^ (*n* = 3),^22^, ^37^, ^62^ YFAS‐C^9^ (*n* = 3),^54^, ^58^, ^59^ and mYFAS^10^ (*n* = 1).^36^

**TABLE 1:** Included study characteristics.

Study	Country	Study type	*N*	Participants	Demographics	Intervention(s)	Month of follow‐up	YFAS and outcome measure (covariates)	Effect of FA	Risk of bias (ROBINS‐I)
Allison et al.^52^	USA	Cohort	300	Adult (18–65 years) bariatric surgery patients (BMI > 35 kg/m^2^)	Age: 40.1 years BMI: 45.9 kg/m^2^ Gender: 87% women Ethnicity: 29% White YFAS count: 2.08 FA positive: 7%	SG RYGB	6, 12	YFAS ➔ %TWL (age, gender, ethnicity, surgery type, baseline BMI, disordered eating scores)	n.s.	Moderate
Ames et al.^38^	USA	Cohort	422	Adult bariatric surgery patients (BMI > 35 kg/m^2^)	Age: 48.4 years BMI: 45.3 kg/m^2^ Gender: 76% women Ethnicity: 93% White YFAS count: 2.41 FA positive: 14%	SG RYGB	12, 24	YFAS ➔ %TWL (Age, gender) Comment: Binge‐eating was assessed, but n.s.	n.s.	Moderate
Bach et al.^35^	Germany	Cohort	26	Adult (18–65 years) bariatric surgery patients (BMI > 35 kg/m^2^)	Age: 41.5 years BMI: 46.4 kg/m^2^ Gender: 65% women Ethnicity: NA YFAS count: 2.54 FA positive: NA	SG RYGB	2, 6	YFAS ➔ %TWL (Feeling of hunger)	↘	Low
Ben‐Porat et al.^63^	Israel	Cohort	54	Adult (18–65 years) bariatric surgery patients (BMI > 35 kg/m^2^)	Age: 32.1 years BMI: 44.9 kg/m^2^ Gender: 100% women Ethnicity: NA YFAS count: 2.43 FA positive: 41%	SG	3, 6, 12	YFAS ➔ %EWL Comment: All patients with food addiction at baseline met binge‐eating criteria	n.s.	Serious
Ben‐Porat et al.^40^	Israel	Cohort	45	Adult (18–65 years) bariatric surgery patients (BMI > 35 kg/m^2^)	Age: 32.4 years BMI: 44.2 kg/m^2^ Gender: 100% women Ethnicity: NA YFAS count: 3.00 FA positive: 40%	SG	3, 6, 12, 24	YFAS ➔ %TWL Comment: Patients with FA positive had higher binge‐eating scores	n.s.	Moderate
Burmeister et al.^32^	USA	Cohort	57	Adults with overweight or obesity (BMI > 27 kg/m^2^)	Age: 47.4 years BMI: 38.2 kg/m^2^ Gender: 68% women Ethnicity: 84% White YFAS count: 3.13 FA positive: NA	BWL (“habit formation, environmental modification, motivation enhancement”; weekly group sessions over 7 weeks)	1.75	YFAS ➔ %TWL Comment: Correlation is n.s. when controlling for binge‐eating	↘	Serious
Camacho‐Barcia et al.^33^	Spain	RCT	448	Adults (55–75 years) with overweight or obesity (BMI > 27 kg/m^2^) and metabolic syndrome	Age: 65.3 years BMI: 32.5 kg/m^2^ Gender: 52% women Ethnicity: 98% White YFAS count: NA FA positive: 6%	BWL (energy‐reduced diet, physical activity promotion; 2 to 3 group sessions per month over 6 years)	12, 24	YFAS ➔ Δ BMI	n.s.	Low
Chao et al.^53^	USA	Cohort	178	Adults (21–65 years) with overweight or obesity (BMI > 30 kg/m^2^) under routine medical care	Age: 44.2 years BMI: 40.9 kg/m^2^ Gender: 88% women Ethnicity: 22% White YFAS count: 2.28 FA positive: 7%	BWL (“lifestyle modification”, energy‐reduced diet; weekly group sessions over 14 weeks)	3.5	YFAS ➔ %TWL (ethnicity, gender, age, baseline BMI)	n.s.	No information
de Almeida et al.^54^	Brazil	Cohort	120	Students (9–11 years) with overweight of a low‐income area	Age: 9.6 years BMI: 1.96 SDS Gender: 53% girls Ethnicity: NA YFAS count: NA FA positive: 33%	BWL (physical activity promotion; weekly school‐based group sessions over 16 months)	16	YFAS‐C ➔ BMI‐SDS (baseline BMI‐SDS, gender, age, location of intervention)	↘	Low
Fielding‐Singh et al.^23^	USA	Cohort	609	Adults (18–50 years) with overweight or obesity (BMI > 28 kg/m^2^)	Age: 39.3 years BMI: 33.4 kg/m^2^ Gender: 57% women Ethnicity: 58% White YFAS count: 2.60 FA positive: 11%	BWL (Energy‐reduced diet; 22 group sessions over 12 months)	12	YFAS ➔ categorical Δ KG: any weight loss vs. weight gain (Ethnicity, social support, disordered eating)	↘	Moderate
Gordon et al.^55^	USA	RCT	182	Adults with overweight and obesity (BMI > 30 kg/m^2^) without contraindications for weight loss	Age: 55.4 years BMI: 36.6 kg/m^2^ Gender: 85% women Ethnicity: 96% White YFAs count: 2.38 FA positive: 13%	BWL (Energy‐reduced diet, physical activity promotion; weekly group sessions over 4 months, telephone or email counseling over 10 months)	4, 10, 22	YFAS ➔ Δ KG (height, ethnicity, baseline problem food consumption)	↘	Low
Guzzardi et al.^60^	Italy	Cohort	36	Women (>18 years) with overweight or obesity (BMI > 25 kg/m^2^)	Age: 36.1 years BMI: 32.8 kg/m^2^ Gender: 100% women Ethnicity: NA YFAS count: NA FA positive: 61%	BWL (energy‐reduced diet; self‐administered over 3 months)	3	YFAS ➔ %TWL	n.s.	Serious
Koball et al.^56^	USA	Cohort	923	Adult (>18 years) bariatric surgery patients	Age: 47.9 years BMI: 45.8 kg/m^2^ Gender: 71% women Ethnicity: 91% White YFAS count: 2.40 FA positive: 14%	RYGB	6, 12	YFAS ➔ %TWL (binge‐eating)	n.s.	Serious
Lent et al.^36^	USA	Cohort	178	Adults with overweight or obesity (BMI > 25 kg/m^2^)	Age: 51.2 years BMI: 36.1 kg/m^2^ Gender: 75% women Ethnicity: 26% White YFAS count: 2.60 FA positive: 15%	BWL (energy‐reduced diet, physical activity promotion, diabetes self‐management; 9 group sessions over 6 months)	6	mYFAS ➔ Δ BMI (treatment arm, baseline weight, gender)	n.s.	Moderate
Mallorquí‐Bagué et al.^37^	Spain	RCT	342	Adults (55–75 years) with overweight or obesity (BMI > 27 kg/m^2^) and metabolic syndrome	Age: 65.2 years BMI: 32.6 kg/m^2^ Gender: 49% women Ethnicity: NA YFAS count: 1.73 FA positive: NA	BWL (Energy‐reduced diet, physical activity promotion, psychosocial support; 2 to 3 group sessions per month over 6 years)	12	YFAS 2.0 ➔ Δ BMI (gender, age, education, baseline BMI, impulsivity)	n.s.	Low
Miller‐Matero et al.^34^	USA	Cohort	101	Adult bariatric surgery patients	Age: 46.0 years BMI: 49.2 kg/m^2^ Gender: 82% women Ethnicity: 65% White YFAS count: 2.19 FA positive: NA	SG RYGB	12	YFAS ➔ %TWL	n.s.	Serious
Nicolau et al.^62^	Spain	Cohort	113	Adult (> 18 years) patients with obesity (BMI > 30 kg/m^2^) after unsuccessful BWL	Age: 45.5 years BMI: 34.4 kg/m^2^ Gender: 69% women Ethnicity: NA YFAS count: NA FA positive: 58%	Pharmacological (0.25 to 1 mg/week semaglutide + self‐administered energy‐reduced diet, physical activity promotion over 4 months)	4	YFAS 2.0 ➔ %TWL Comment: Patients with FA positive reported more binge‐eating episodes	n.s.	Serious
Pepino et al.^57^	USA	Cohort	44	Adult bariatric surgery patients with obesity	Age: 42.8 years BMI: 48.0 kg/m^2^ Gender: 89% women Ethnicity: NA YFAS count: 2.95 FA positive: 32%	SG RYGB LAGB	5	YFAS ➔ %TWL	n.s.	Moderate
Perez et al.^22^	Spain	Cohort	110	Adult (18–65 years) bariatric surgery patients (BMI > 35 kg/m^2^)	Age: 47.3 years BMI: 46.0 kg/m^2^ Gender: 76% women Ethnicity: NA YFAS count: 2.90 FA positive: 26%	BWL (energy‐reduced diet, physical activity promotion; 4 individual sessions over 6 months)	6	YFAS 2.0 ➔ %TWL (gender, age, weight at baseline)	↘	Low
Ribeiro et al.^61^	Portugal	Cohort	116	Adult bariatric surgery patients	Age: 43.3 years BMI: 43.1 kg/m^2^ Gender: 85% women Ethnicity: NA YFAS count: 2.50 FA positive: 18%	SG RYGB	11, 18	YFAS ➔ %TWL (BMI, baseline age, gender, personal history of type 2 diabetes mellitus, surgical center, surgery type)	n.s.	Moderate
Sawamoto et al.^64^	Japan	Cohort	86	Women with overweight (BMI > 25 kg/m^2^) without physical impairment	Age: 48.3 years BMI: 31.2 kg/m^2^ Gender: 100% women Ethnicity: NA YFAS count: 2.08 FA positive: NA	BWL (Cognitive behavioral group therapy, “guided lifestyle change”;40 group sessions over 7 months)	12, 24	YFAS ➔ categorical: more or less than 10% TWL Comment: Binge‐eating assessed, but correlation not reported	n.s.	Moderate
Sevinçer et al.^65^	Turkey	Cohort	166	Adult (> 18 years) bariatric surgery patients (BMI > 35 kg/m^2^)	Age: 36.1 years BMI: 47.0 kg/m^2^ Gender: 77% women Ethnicity: NA YFAS count: 3.75 FA positive: 58%	SG	6, 12	YFAS ➔ %EWL	n.s.	Low
Testa et al.^41^	Italy	Cohort	69	Adult bariatric surgery patients (BMI > 35 kg/m^2^)	Age: 42.6 years BMI: 43.6 kg/m^2^ Gender: 87% women Ethnicity: NA YFAS count: 3.02 FA positive: NA	SG	12	YFAS ➔ categorical: more or less than 50% EWL (Age, baseline BMI, metabolic diseases, impulsiveness) Comment: Binge‐eating assessed, but n.s.	n.s.	Low
Vidmar et al.^58^	USA	Cohort	18	Adolescent (12–18 years) patients in a weight management clinic	Age: 14.4 years BMI: 2.2 SDS Gender: 72% girls Ethnicity: 17% White YFAS count: NA FA positive: 100%	BWL (addiction‐based, app‐mediated intervention; 2 clinical visits, 5 text messages per week and weekly phone sessions over 6 months)	1, 3, 6	YFAS‐C ➔ BMI‐SDS (controlled for baseline)	n.s.	Moderate
Vidmar et al.^59^	USA	RCT	161	Adolescents (14–18 years) with obesity (BMI‐SDS > 95th percentile)	Age: 16.0 years BMI: 2.2 SDS Gender: 65% girls Ethnicity: 40% White YFAS count: NA FA positive: 35%	BWL (addiction‐based app [see above] vs. energy‐reduced diet, physical activity promotion [6 individual sessions]; 6 months)	12, 24	YFAS‐C ➔ % lost BMI‐SDS (type of intervention, stress, binge eating, gender) Comment:FA predicted weight in interaction with gender	n.s.	Low

Abbreviations: *N*, total number of study patients; BWL, behavioral weight loss; SG, sleeve gastrectomy/vertical sleeve gastrectomy; RYGB, Roux‐en‐Y bypass; LAGB, laparoscopic adjustable gastric band; %TWL, percent total weight loss; %EWL, percent excess weight loss; Δ, raw differences between pre‐ and post‐weight status assessment; BMI‐SDS, age‐adjusted BMI standard deviation scores; (m)YFAS(‐C) (2.0), (modified) Yale Food Addiction Scale (for Children) (updated based on DSM‐5); NA, data not available; n.s., not significant; ↗, more FA symptoms or FA positive diagnosis predicted increased weight loss; ↘, higher FA symptom count or FA positive diagnosis predicted decreased weight loss.

The 25 studies included 4904 individuals (3475 women, 1429 men). Three studies included children (*n* = 299) with the remaining including only adults (*n* = 4605). Four studies included women only (*n* = 221).^40^, ^60^, ^63^, ^64^ The mean age across all studies was 41.4 (SD = 13.39; *M*_adults_ = 45.4, SD = 8.65, *M*_children_ = 13.3, SD = 3.33), the adults' average BMI was 40.82 (SD = 6.04; the BMI‐SDS average in studies with children was 2.12, SD = 0.14). Average YFAS symptom count across studies was 2.58 (SD = 0.46), and the average FA prevalence was 31%.

### 3.3 Risk of bias

The majority of studies achieved a low (*n* = 9)^22^, ^33^, ^37^, ^38^, ^41^, ^54^, ^55^, ^59^, ^65^ or moderate (*n* = 9)^23^, ^36^, ^38^, ^40^, ^52^, ^57^, ^58^, ^61^, ^64^ risk‐of‐bias score on the ROBINS‐I (see Table S1). Six studies were classified as serious risk (due to failing to control for known confounds),^32^, ^34^, ^56^, ^60^, ^62^, ^63^ and one study^53^ did not provide enough information in one category of bias judgment. We included the risk‐of‐bias score (coded 0, 1, 2 for low, moderate, and serious risk, respectively) as a predictor in meta‐regression in the syntheses below.

### 3.4 Results of syntheses

#### 3.4.1 Pre‐intervention YFAS symptom count and weight loss correlation

**FIGURE 2:**
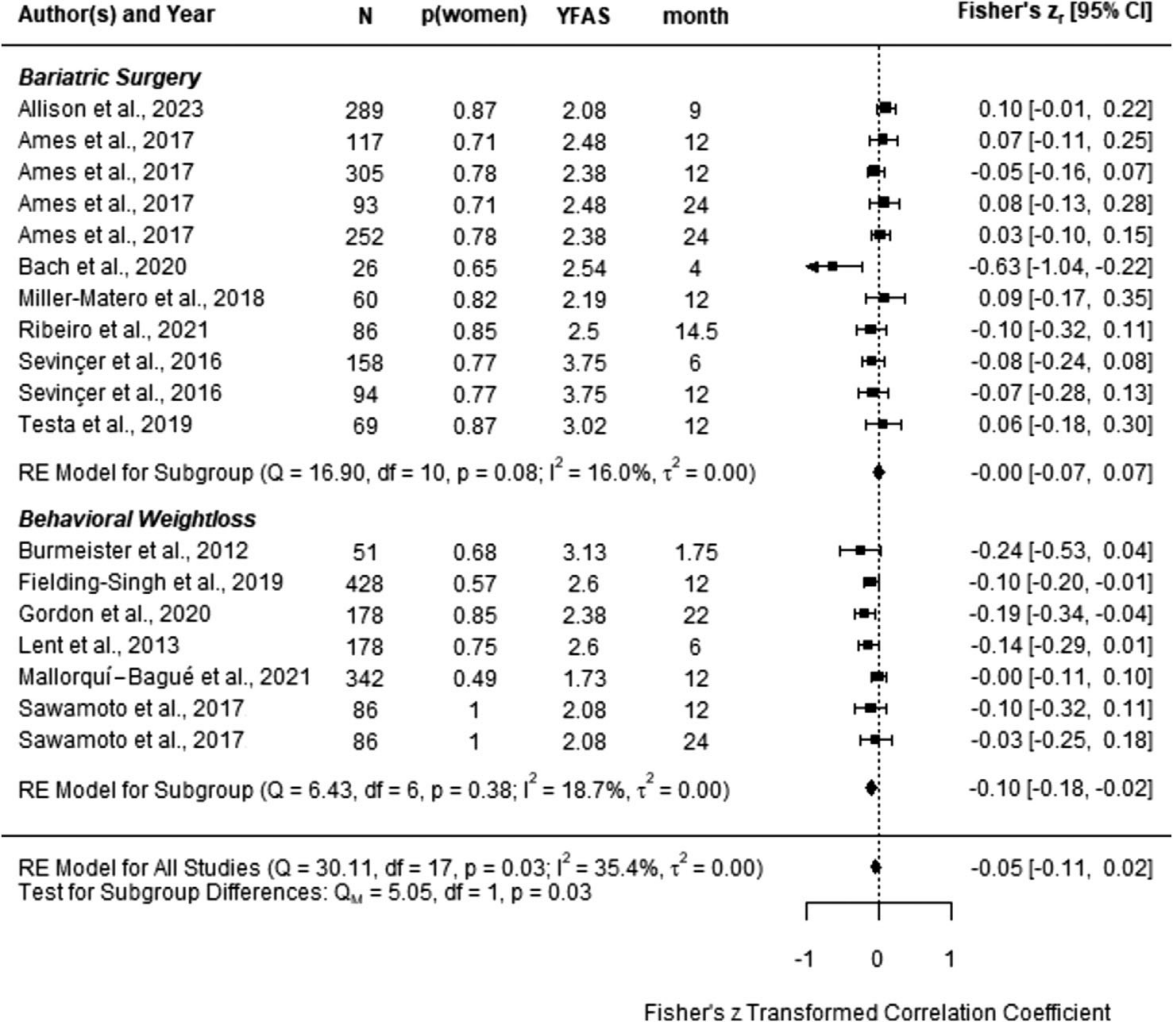
Forest plot of Fisher's z‐transformed correlation estimates between pre‐intervention YFAS symptom count and weight loss outcomes from random‐effect models with cluster‐robust variance estimation. More negative correlations indicate lower weight loss with higher level of pre‐intervention food addiction. N, analyzed sample; p (women), relative frequency of women in the sample; YFAS, Yale Food Addiction Scale symptom count; CI, confidence interval.

**FIGURE 3:**
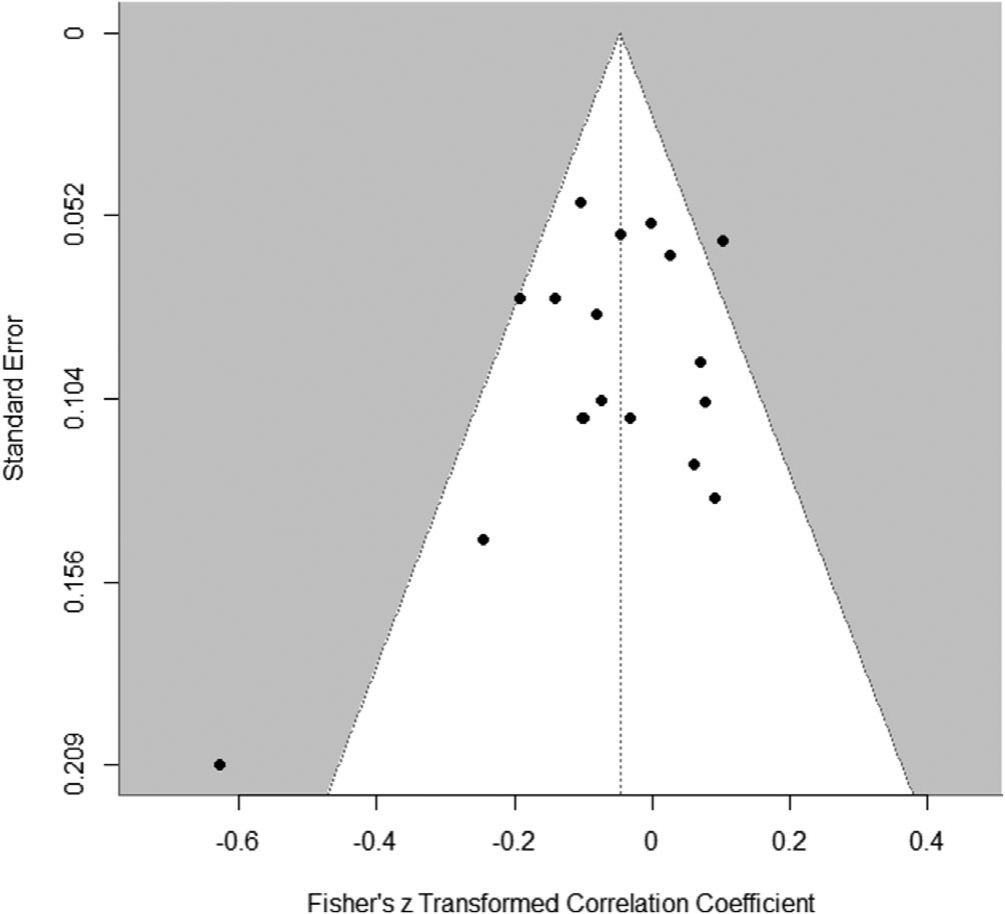
Funnel plot of effect sizes included in the meta‐analyses of correlation estimates between pre‐intervention FA symptom count and weight loss.

The pooled correlation estimate between pre‐intervention YFAS symptom count and weight loss across 13 studies (*k* = 18) was negative, but not significant, *z* = −0.05, 95% CI [−0.11; 0.02], *t* (12) = − 1.61, *p* = 0.13 (see Figure 2). Visual inspection of the funnel plot (Figure 3) showed some asymmetry, but the Egger's test suggested low potential for publication bias, *p* = 0.14.

The heterogeneity of effect sizes was moderate, *I* ^2^ = 35.41%, and we next explored systematic variations in several univariate meta‐regressions (see Table S2). According to tests of moderation, correlations did not differ by the follow‐up interval, *p* = 0.34; patient gender, *p* = 0.64; pre‐intervention BMI, *p* = 0.13; pre‐intervention YFAS symptom count, *p* = 0.10; ethnicity, *p* = 0.94; or study risk of bias (coded 0, 1, 2 for low, moderate, and serious risk), *p* = 0.46. However, effect sizes differed by the type of intervention, *p* = 0.03, with higher YFAS symptom count predicting lower weight loss only for behavioral weight loss interventions, z = −0.10, 95% CI [−0.17; −0.03], *t* (11) = − 3.18, *p* = 0.009, but not after surgery, z = −0.00, 95% CI [−0.06; 0.06], *t* (11) = − 0.01, *p* = 0.99.

#### 3.4.2 Pre‐intervention YFAS diagnosis groups and weight loss

**FIGURE 4:**
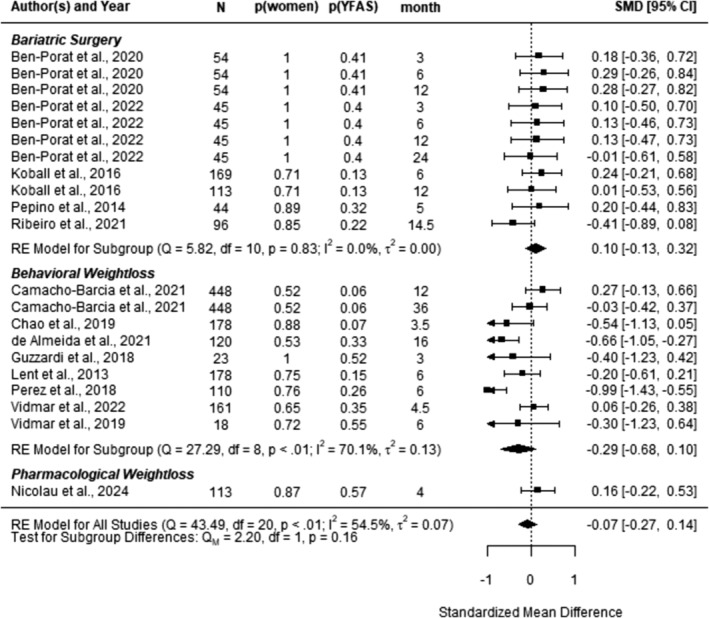
Forest plot of standardized mean differences of weight loss outcomes between individuals with pre‐intervention FA positive and FA negative from random‐effect models with cluster‐robust variance estimation. More negative values indicate lower weight loss in FA positive compared to FA negative. N, analyzed sample; p (women), relative frequency of women in the sample; p (YFAS), relative frequency of food addition positive individuals based on the Yale Food Addiction Scale in the sample; CI, confidence interval.

**FIGURE 5:**
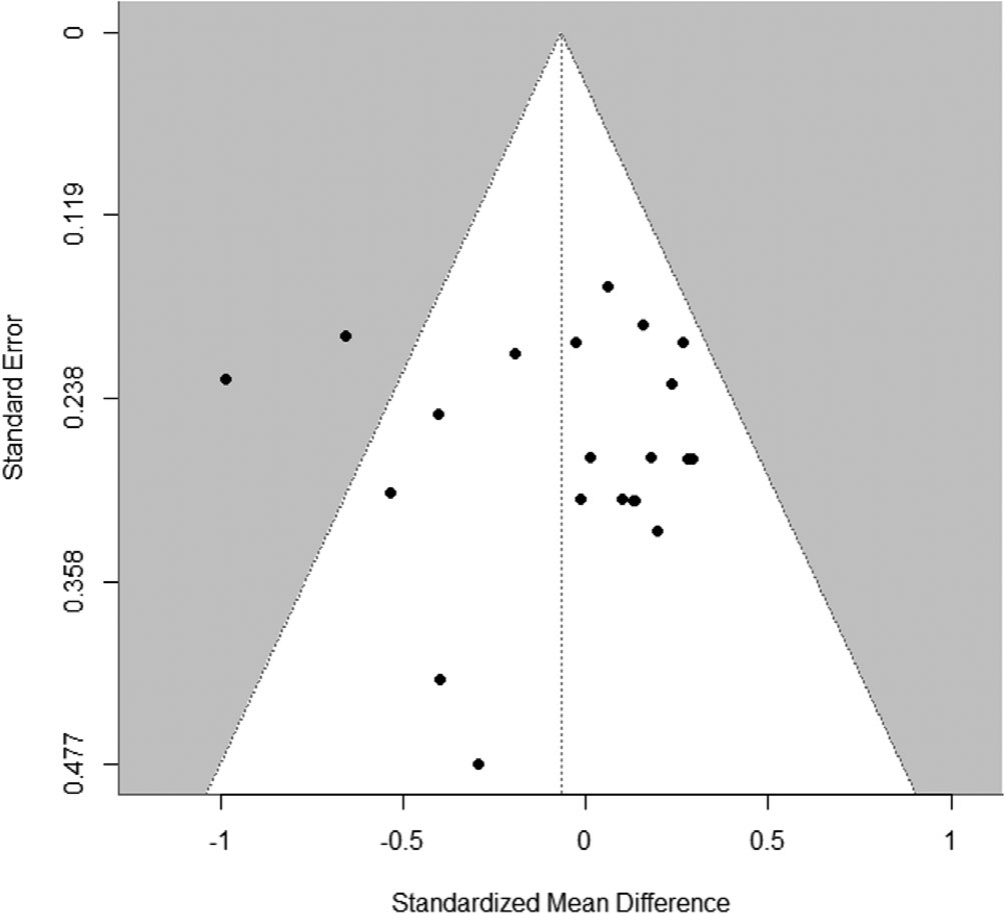
Funnel plot of effect sizes included in the meta‐analyses of standardized mean differences of weight loss between individuals with pre‐intervention FA positive and food addiction negative.

The pooled differences of weight loss between individuals with pre‐intervention FA positive and FA negative across 14 studies (*k* = 21) were negative (i.e., favoring FA negative over FA positive in terms of weight loss), but not significant, *SMD* = −0.07, 95% CI [−0.27; 0.14], *t* (13) = − 0.72, *p* = 0.48 (see Figure 4). Visual inspection of the funnel plot (Figure 5) revealed some asymmetry, but the Egger's test was not significant, *p* = 0.98.

The heterogeneity of effect sizes was moderate, *I* ^2^ = 54.46%. Explorations of systematic variations using meta‐regressions (see Table S3) found no differences based on the type of intervention (surgery vs. behavioral weight loss intervention; the pharmacological intervention was excluded due to the insufficient number of effect sizes for group comparisons), *p* = 0.16; the follow‐up interval, *p* = 0.78; patient gender, *p* = 0.42; pre‐intervention BMI, *p* = 0.90; YFAS symptom count, *p* = 0.86; age group, *p* = 0.48; or study risk‐of bias coding, *p* = 0.15. However, ethnicity moderated the difference, *p* = 0.04. More diverse samples tended to show less weight loss among patients with FA positive compared to FA negative, *SMD* = −0.29, 95% CI [−0.70; 0.12], *t* (5) = − 1.79, *p* = 0.14. This estimate changed by 0.47, 95% CI [0.01; 0.91] in homogenous White samples, *t* (5) = 2.65, *p* = 0.04.

## 4 DISCUSSION

This systematic review with meta‐analysis estimated the prognostic effect of FA diagnosis and symptom count on weight loss intervention outcomes in individuals with overweight or OB without a medically diagnosed eating disorder. The prevalence of FA across the included 25 studies prior to any intervention was comparable to rates in previous investigations.^16^ Neither a positive FA diagnosis nor the FA symptom count was directly associated with weight loss following bariatric surgery or after pharmacological intervention. However, higher FA symptom count (but not a FA‐positive diagnosis) predicted lower weight loss after behavioral weight loss interventions, and FA diagnosis was associated with weight loss for important subgroups (see below). Thus, we found limited support for a negative prognostic effect of FA on weight loss intervention outcomes.

The predictive effect of FA symptom count on weight loss following behavioral interventions could suggest that more severe and dysfunctional forms of FA could lead to poorer treatment responses.^66^ Compared to surgery and pharmacological interventions, behavioral interventions often take place over several weeks or months and thus require continued effort to adhere to dieting plans, exercising, and other lifestyle changes. These efforts could be counteracted by experiencing loss of control over eating and persistent cravings for highly palatable foods that are characteristic of FA. However, whether this is actually the case and at what levels FA might hamper the overall outcomes of behavioral OB interventions requires further investigation. Indeed, we did not find a FA diagnosis to be associated with less weight loss following behavioral interventions, although the studies included in this analysis exclusively relied on DSM‐IV‐based FA criteria. Since severity grades were only included in the updated YFAS 2.0 in addition to the binary diagnosis,^11^ future syntheses will likely reveal if the prognostic role of FA could be linked to clinical severity.

Moreover, the detrimental effects of FA could depend on further moderating conditions. Specifically, our secondary analyses found differences related to the ethnicity of participants. More diverse samples with FA‐positive seeking weight‐loss treatment tended to show less weight loss compared to individuals with FA‐negative. Although FA symptoms appear more prevalent, for example, in White compared to Black bariatric surgery candidates,^67^ Black patients in the United States also experience lower weight loss.^68^ This effect could be due to differences in lower access to healthy foods in mixed ethnicity compared to predominantly White communities,^69^ which could also serve as the basis for the more detrimental effect of FA observed for more diverse samples here. It has been argued that aspects of the FA concept may be culture‐dependent,^14^ suggesting that effects of ethnicity should be interpreted with caution. Yet, it must be noted that the YFAS scale has been extensively validated both quantitively and qualitatively across diverse samples.^16^, ^19^, ^70^, ^71^

Overall, our findings might suggest that FA plays a specific but limited role in the outcome of OB interventions. In many cases, FA could be a frequent co‐morbidity of OB,^16^ as altered hedonic mechanisms of body weight regulation in OB may be associated with higher FA symptoms.^72^ Eating disorders, specifically (sub‐threshold) binge‐eating disorder, might also explain the co‐morbidity of FA and OB^6^ in some cases as well as why a positive FA diagnosis or pronounced FA symptoms were not broadly associated with OB intervention outcomes. Thus, FA may carry several implications especially for designing targeted interventions for specific subgroups. At the same time, it is important to acknowledge that OB interventions may also reduce FA symptoms^73^ and, through this reduction, indirectly affect OB intervention outcomes. Unfortunately, only a few of the identified studies tracked and associated changes in FA with changes in body weight longitudinally,^55^ rendering a systematic analysis of such patterns infeasible at the moment.

### 4.1 Limitations

Interpreting the present findings is subject to limitations. First, we only considered effects of FA in individuals without a medically diagnosed eating disorder. While this was essential for exploring the unique contribution of FA to weight loss intervention outcomes, we can neither exclude that individuals with FA exhibited subthreshold ED symptoms, nor that we limited our ability to detect other relevant effects by omitting more severe FA cases from the analysis (in a similar vein, FA could be present in many studies on binge eating without being assessed). Indeed, not all studies controlled for binge‐eating symptoms, which, in some studies, was reported to qualify the effects of FA on weight loss outcomes.^74^ The studies also differed in their criteria of including only individuals with OB or also with overweight, which could also have affected our results (note, however, that the pre‐intervention BMI did not emerge as a moderator in our analysis). We also did not contact authors for unreported analyses, which could have helped to increase the power of our meta‐analyses further.

Second, we included mostly studies that used the YFAS version based on the now outdated and stricter DSM‐IV criteria, which may have reduced the sensitivity to detect further associations. Future studies are warranted to replicate the present findings using the updated YFAS 2.0 version and explore the prognostic role of specific FA symptoms (e.g., craving).

Third, we thus far only considered weight loss as an intervention outcome, but we did not evaluate intervention adherence or drop out rates^23^; this must be kept in mind while interpreting the results of the present analysis. Since drop outs could significantly alter the overall success of weight loss in a FA positive compared to a FA negative group, further extending the considered range of outcomes is essential.

Finally, we must note that FA has been linked to greater symptoms of mental health problems^75^, ^76^ and these, in turn, have been related to poorer outcomes in weight loss programs.^77^, ^78^ It thus remains an unanswered question how addressing FA in treatment might facilitate the outcomes of weight loss interventions, or whether addiction‐based weight loss interventions might be more effective than other types of interventions, especially in the case of higher FA severity. Further studies could examine, for example, the role of FA as a moderator between mental health problems and poor weight loss outcomes to determine whether the results described in other similar populations, such as those with binge‐eating disorder,^79^, ^80^, ^81^ can be replicated.

## 5 CONCLUSION

FA remains an issue of ongoing controversy. This is the first systematic review with meta‐analysis to examine the prognostic role of FA for the success of weight loss interventions. The analysis found limited support for a detrimental effect of FA. Our results suggest that FA could predict lower weight loss following behavioral weight loss interventions, and in ethnically diverse populations. It remains an unanswered question whether FA might have an indirect role in the outcome of other weight loss interventions. Further studies could examine, for example, the role of FA as a moderator between increased mental health problems and poor weight loss outcome.

## CONFLICT OF INTEREST STATEMENT

FFA received consultancy honoraria from Novo Nordisk and editorial honoraria as EIC from Wiley. The rest of the authors declare no conflict of interest. The funders had no role in the design of the study; in the collection, analyses, or interpretation of data; in the writing of the manuscript; or in the decision to publish the results.

## Supporting information


**Table S1** Rating results for the Cochrane risk of bias tool in non‐randomized studies and interventions (ROBINS‐I).
**Table S2** Meta‐regression results for testing the influence of study and participant variables on Fisher's z‐transformed correlation estimates between pre‐intervention YFAS symptom count and weight loss outcomes.
**Table S3** Meta‐regression results for testing the influence of study and participant variables on standardized mean differences of weight loss outcomes between individuals with pre‐intervention FA positive and FA negative.
