# Supplementary material for: The prognostic role of food addiction for weight loss treatment outcomes in individuals with overweight and obesity: A systematic review and meta‐analysis
Source: Obes Rev. 2024 Oct 16;26(2):e13851. doi: 10.1111/obr.13851 (PMC11711077; doi:10.1111/obr.13851)
Supplement: Supplementary file 1 — Table S1 Rating results for the Cochrane risk of bias tool in non‐randomized studies and interventions (ROBINS‐I). Table S2 Meta‐regression results for testing the influence of study and participant variables on Fisher's z‐transformed correlation estimates between pre‐intervention YFAS symptom count and weight loss outcomes. Table S3 Meta‐regression results for testing the influence of study and participant variables on standardized mean differences of weight loss outcomes between individuals with pre‐intervention FA positive and FA negative. [file OBR-26-e13851-s001.pdf]

Supporting Information

Title:

The Prognostic Role of Food Addiction for Weight Loss Treatment Outcomes in Individuals with Overweight and Obesity: A Systematic Review and Meta-Analysis

Authors:

Georg Halbeisen[1], Marie Pahlenkemper[1], Luisa Sabel[1], Candice Richardson[2], Zaida Agüera[3,4,5,6], Fernando Fernandez-Aranda[5,6,7,8], Georgios Paslakis[1]

1 University Clinic for Psychosomatic Medicine and Psychotherapy, Medical Faculty, Campus East-Westphalia, Ruhr-University Bochum, Germany
2 Temerty Faculty of Medicine, University of Toronto, Canada
3 Departament d'Infermeria de Salut Pública, Salut Mental i Materno-Infantil, Escola d'Infermeria, Facultat de Medicina i Ciències de la Salut, Universitat de Barcelona, Spain
4 Research group in Mental health, Psychosocial and Complex Nursing Care (NURSEARCH), Facultat de Medicina i Ciències de la Salut, Universitat de Barcelona, Spain
5 CIBER Fisiopatología Obesidad y Nutrición (CIBERobn), Instituto de Salud Carlos III, Madrid, Spain
6 Psychoneurobiology of Eating and Addictive Behaviors Group, Neurosciences Programme, Bellvitge Biomedical Research Institute (IDIBELL), L'Hospitalet de Llobregat, Barcelona, Spain
7 Eating Disorders Unit, Clinical Psychology Unit, University Hospital of Bellvitge, L'Hospitalet de Llobregat, Spain
8 Department of Clinical Sciences, School of Medicine and Health Sciences, University of Barcelona, Spain.

Correspondence:

Georg Halbeisen, University Clinic for Psychosomatic Medicine and Psychotherapy, Medical Faculty, Campus East-Westphalia, Ruhr-University Bochum, Virchowstr. 65, 32312 Luebbecke, Germany. Email: georg.halbeisen@rub.de

Electronic Database Search Keywords

PubMed

((Food Addiction [mh] OR Compulsive Eating [mh] OR Addictive Behaviour [mh])

OR

((food addiction) OR (compulsive eating) OR (compulsive) OR (addictive behavio*) OR (eating addiction) OR (Yale food addiction scale) OR (YFAS)))

AND

((Obesity [mh] OR Body Weight [mh] OR Weight [mh] OR Body Weight change [mh] OR Weight Loss [mh] OR Weight Reduction [mh] OR Body Weight Trajectory [mh] OR Weight Change trajectory [mh] OR Weight Loss Trajectories [mh] OR Body Mass Index [mh])

OR

((obes*) OR (obese patient*) OR (body weight) OR (weight) OR (overweight) OR (body weight change*) OR (weight loss) OR (weight reduction) OR (body weight trajector*) OR (weight change trajector*) OR (body composition) OR (weight loss trajector*) OR (body mass index) OR (bmi)))

AND

((Obesity Management [mh] OR Bariatric surgery [mh] OR Bariatric surgery procedures [mh] OR Gastric Bypass [mh] OR Gastroplasty [mh] OR Stomach Stapling [mh] OR Weight Reduction Diet [mh] OR Weight loss diet [mh] OR Cognitive Behavioural Therapy [mh] OR Drug therapy [mh] OR Physical Exercise [mh] OR Obesity Management [mh] OR Weight Reduction Program [mh])

OR

((obesity management) OR (bariatric) OR (bariatric surger*) OR(obesity surger*) OR (Bariatric surgery procedure*) OR (gastric bypass) OR (gastroplasty) OR (stomach stapling) OR (weight reduction diet*) OR (quality of life) OR (mental health) OR (physical health) OR (dialectical behavior therapy) OR (self-management) OR (cognitive behavioural therapy) OR (cognitive behavioral therapy) OR (cbt) OR (pharmacological intervention*) OR (non-pharmacological intervention*) OR (orlistat) OR (sitagliptin) OR (metformin) OR (topiramate) OR (drug therap*) OR (physical exercise) OR (lifestyle change*) OR (treatment outcome) OR (treatment response) OR (therapy response) OR (dietetic intervention) OR (weight reduction program*)))

PsychInfo (via Ebsco with fulltext search)

((MA Food Addiction OR MA Compulsive Eating OR MA Addictive Behaviour)

OR

((food addiction) OR (compulsive eating) OR (compulsive) OR (addictive behavio*) OR (eating addiction) OR (Yale food addiction scale) OR (YFAS)))

AND

((MA Obesity OR MA Body Weight OR MA Weight OR MA Body Weight change OR MA Weight Loss OR MA Weight Reduction OR MA Body Weight Trajectory OR MA Weight Change trajectory OR MA Weight Loss Trajectories OR MA Body Mass Index)

OR

((obes*) OR (obese patient*) OR (body weight) OR (weight) OR (overweight) OR (body weight change*) OR (weight loss) OR (weight reduction) OR (body weight trajector*) OR (weight change trajector*) OR (body composition) OR (weight loss trajector*) OR (body mass index) OR (bmi)))

AND

((MA Obesity Management OR MA Bariatric surgery OR MA Bariatric surgery procedures OR MA Gastric Bypass OR MA Gastroplasty OR MA Stomach Stapling OR MA Weight Reduction Diet OR MA Weight loss diet OR MA Cognitive Behavioural Therapy OR MA Drug therapy OR MA Physical Exercise OR MA Obesity Management OR MA Weight Reduction Program)

OR

((obesity management) OR (bariatric) OR (bariatric surger*) OR(obesity surger*) OR (Bariatric surgery procedure*) OR (gastric bypass) OR (gastroplasty) OR (stomach stapling) OR (weight reduction diet*) OR (quality of life) OR (mental health) OR (physical health) OR (dialectical behavior therapy) OR (self-management) OR (cognitive behavioural therapy) OR (cognitive behavioral therapy) OR (cbt) OR (pharmacological intervention*) OR (non-pharmacological intervention*) OR (orlistat) OR (sitagliptin) OR (metformin) OR (topiramate) OR (drug therap*) OR (physical exercise) OR (lifestyle change*) OR (treatment outcome) OR (treatment response) OR (therapy response) OR (dietetic intervention) OR (weight reduction program*)))

Web of Science

(ALL=(food addiction) OR ALL=(compulsive eating) OR ALL=(compulsive) OR ALL=(addictive behavio*) OR ALL=(eating addiction) OR ALL=(Yale food addiction scale) OR ALL=(YFAS))

AND

(ALL=(obes*) OR ALL=(obese patient*) OR ALL=(body weight) OR ALL=(weight) OR ALL=(overweight) OR ALL=(body weight change*) OR ALL=(weight loss) OR ALL=(weight reduction) OR ALL=(body weight trajector*) OR ALL=(weight change trajector*) OR ALL=(body composition) OR ALL=(weight loss trajector*) OR ALL=(body mass index) OR ALL=(bmi))

AND

(ALL=(obesity management) OR ALL=(bariatric) OR ALL=(bariatric surger*) OR ALL=(obesity surger*) OR ALL=(Bariatric surgery procedure*) OR ALL=(gastric bypass) OR ALL=(gastroplasty) OR ALL=(stomach stapling) OR ALL=(weight reduction diet*) OR ALL=(quality of life) OR ALL=(mental and physical health) OR ALL=(dialectical behavior therapy) OR ALL=(self-management) OR ALL=(cognitive behavioural therapy) OR ALL=(cognitive behavioral therapy) OR ALL=(cbt) OR ALL=(pharmacological intervention*) OR ALL=(orlistat) OR ALL=(sitagliptin) OR ALL=(metformin) OR ALL=(topiramate) OR ALL=(physical exercise) OR ALL=(lifestyle change*) OR ALL=(treatment outcome) OR ALL=(treatment response) OR ALL=(therapy response) OR ALL=(dietetic intervention) OR ALL=(weight reduction program*))

Table S1

Rating results for the Cochrane risk of bias tool in non-randomized studies and interventions (ROBINS-I).

Study	Confounding	Participant Selection	Intervention Classification	Deviation from Intervention	Missing Data	Outcome Measurement	Reporting	Overall Rating
Allison et al. 2023	moderate	low	low	low	low	low	low	moderate
Ames et al. 2017	moderate	low	low	low	low	low	low	moderate
Bach et al. 2021	low	low	low	low	low	low	low	low
Ben-Porat et al. 2021	serious	low	low	low	low	low	low	serious
Ben-Porat et al. 2022	moderate	low	low	low	low	low	low	moderate
Burmeister et al. 2013	serious	low	low	low	low	low	low	serious
Camacho-Barcia et al. 2021	low	low	low	low	low	low	low	low
Chao et al. 2019	low	low	low	no information	low	low	low	no information
deAlmeida et al. 2021	low	low	low	low	low	low	low	low
Fielding-Singh et al. 2019	moderate	low	low	low	moderate	low	low	moderate
Ribeiro et al. 2021	low	low	low	low	low	moderate	low	moderate
Gordon et al. 20et al. 20	low	low	low	low	low	low	low	low
Guzzardi et al. 2018	serious	low	low	low	low	low	low	serious
Koball et al. 2016	serious	low	low	low	low	low	low	serious
Lent et al. 2014	moderate	low	low	low	low	low	low	moderate
Mallorquí-Bagué et al. 2021	low	low	low	low	low	low	low	low
Miller-Matero et al. 2018	serious	low	low	low	low	low	low	serious
Nicolau et al. 2024	serious	low	low	low	low	low	moderate	serious
Pepino et al. 2014	low	low	low	low	moderate	low	low	moderate
Pérez et al. 2018	low	low	low	low	low	low	low	low
Sawamoto et al. 2017	low	low	low	low	low	moderate	low	moderate
Sevinçer et al. 2016	low	low	low	low	low	low	low	low
Testa et al. 20et al. 20	low	low	low	low	low	low	low	low
Vidmar et al. 2019	low	moderate	low	low	low	low	low	moderate
Vidmar et al. 2022	low	low	low	low	low	low	low	low

Table S2

Meta-regression results for testing the influence of study and participant variables on Fisher's z-transformed correlation estimates between pre-intervention YFAS symptom count and weight loss outcomes.

Moderator	N	k	Estimate	LL	UL	Test Statistics
			Study Variables			
Intervention	13	18				F(2, 11) = 5.05, p = .03
BS			-0.00	-0.06	0.06	t(11) = -0.00, p = .99
BWL			-0.10	-0.17	-0.03	t(11) = -3.18, p = .009
Follow-up Interval	13	18				F(1, 11) = 0.98, p = .34
intercept (0 months)			-0.11	-0.25	0.04	t(11) = -1.61, p = .14
per month increase			0.00	-0.01	0.01	t(11) = 0.99, p = .34
ROBINS-I	13	18				F(1, 11) = 0.58, p = .46
intercept (low bias)			-0.08	-0.18	0.02	t(14) = -1.68, p = .12
per unit increase			0.04	-0.07	0.15	t(14) = 0.76, p = .46
			Participant Variables			
Pre-intervention BMI	13	18				F(1, 11) = 2.64, p = .13
intercept			-0.29	-0.63	0.04	t(11) = -1.94, p = .08
per unit increase			0.01	-0.00	0.02	t(11) = 1.63, p = .13
Gender	13	18				F(1, 11) = 0.23, p = .64
intercept (0% women)			-0.11	-0.39	0.18	t(11) = -0.83, p = .42
100% women			0.08	-0.29	0.45	t(11) = 0.48, p = .64
Ethnicity	7	10				F(1, 5) = 0.01, p = .94
intercept (0% White)			-0.02	-0.37	0.32	t(5) = -0.18, p = .86
100% White			-0.02	-0.47	0.43	t(7) = -0.08, p = .94
YFAS symptom count	13	18				F(1, 11) = 3.26, p = .10
intercept (0 symptoms)			0.10	-0.09	0.29	t(11) = 1.12, p = .28
per unit increase			-0.06	-0.13	0.01	t(11) = -1.80, p = .10

Note. We do not report effects of age group as studies with children did not report symptom count correlations. N = number of studies included in the respective regression; k = number of effect sizes; LL = 95 % confidence interval lower level; UL = 95 % confidence interval upper level.

Table S3

Meta-regression results for testing the influence of study and participant variables on standardized mean differences of weight loss outcomes between individuals with pre-intervention FA positive and FA negative.

Moderator	N	k	Estimate	LL	UL	Test
			Study Variables			
Intervention*	13	20				F(2, 11) = 2.20, p = .26
BS			0.10	-0.07	0.26	t(11) = 1.33, p = .21
BWL			-0.28	-0.65	0.10	t(11) = -1.62, p = .13
Pharmacological			0.16			
Follow-up Interval	14	21				F(1, 12) = 0.08, p = .78
intercept (0 months)			-0.05	-0.33	0.23	t(12) = -0.38, p = .71
per month increase			0.00	-0.02	0.01	t(12) = 0.29, p = .78
ROBINS-I	13	20				F(1, 11) = 2.45, p = .15
intercept (low bias)			-0.25	-0.70	0.20	t(11) = -1.21, p = .25
per unit increase			0.20	-0.08	0.47	t(11) = 1.56, p = .15
			Participant Variables			
Pre-intervention BMI	11	18				F(1, 9) = 0.02, p = .90
intercept			0.06	-1.37	1.48	t(9) = 0.09, p = .93
per unit increase			-0.00	-0.04	0.04	t(9) = -0.13, p = .90
Age Group	14	21				F(2, 12) = 0.78, p = .48
adult			-0.03	-0.25	0.19	t(12) = -0.30, p = .77
children			-0.28	-0.79	0.22	t(12) = -1.21, p = .25
Gender	14	21				F(1, 12) = 0.69, p = .42
intercept (0% women)			-0.44	-1.49	0.62	t(12) = -0.90, p = .39
100% women			0.46	-0.74	1.65	t(12) = 0.83, p = .42
Ethnicity	7	9				F(1, 5) = 7.01, p = .04
intercept (0% White)			-0.29	-0.70	0.12	t(5) = -1.79, p = .13
100% White			0.47	0.01	0.92	t(5) = 2.65, p = .04
YFAS symptom count	8	14				F(1, 6) = 0.03, p = .86
intercept (0 symptoms)			0.19	-3.06	3.44	t(6) = 0.14, p = .89
per unit increase			-0.09	-1.33	1.14	t(6) = -0.18, p = .86

Note. *The pharmacological intervention was excluded due to the insufficient number of effect sizes for a group comparison; N = number of studies included in the respective regression; k = number of effect sizes; LL = 95 % confidence interval lower level; UL = 95 % confidence interval upper level.
